# ‘I Was Treated Like a Bishop in a Vicarage’: Lotta Svärd and the National Socialist Cult of Masculinity as Portrayed by a Lotta Matron and a Voluntary Military Nurse

**DOI:** 10.1093/shm/hkaf060

**Published:** 2026-03-09

**Authors:** Elina Sopo

**Affiliations:** School of History, Culture and Arts Studies, University of Turku, Pori, Finland

**Keywords:** Germany, masculinity, aesthetics of politics, military nursing, gender relations

## Abstract

This study explores the Finnish voluntary auxiliary paramilitary organisation, Lotta Svärd, which was engaged in wartime nursing during Operation Barbarossa. First, it analyses the public image of Lotta from the Medical Division and illustrates how this became charged with political value, mirroring an identical phenomenon in 1930s Germany. Then, structuring arguments from a regional archive, it showcases the reception of a Lotta district leader by German medical officers, nursing and women’s organisations, and finally reflects the experience of a military nurse in a Wehrmacht hospital in Finland. The interest in Lotta Svärd was planted and the character was discovered by Alfred Rosenberg. The Spartanly simple, bureaucratically conscientious organisation offered a cultural frame in which political authority defined itself. Lottas mirrored the ‘moral’, utopian ideal of national socialist leadership, reinforced the masculine image of German troops, but were ultimately precious as role models by the Third Reich’s political thought, aesthetics and bureaucracy.

In a short note of thanks, read on a summer evening in Potsdam on 1 June 1944, ‘*Frau Oberin*’^1^ or ‘*Lotta Führerin*’, Hilda B. expressed her gratification to *Reichsführer* Heinrich Himmler for the spiritual and physical re-fuelling vacation offered to the Lotta Svärd organisation. The dinner, given by the *Schutzstaffel* (SS) leadership, was served at the favourite *Nationalsozialistische Deutsche Arbeitpartei* (NSDAP) party hangout Regattahaus where, in the past, for example, Joseph Göbbels had held political negotiations in 1933.^2^ In her speech, Hilda expressed how wonderful it would be if Mr *Reichsführer* were to appear at the Lotta’s dwelling place in south-eastern Bavaria sometime during their almost 2-month stay in Germany.^3^ The country house in which Hilda was staying was the same estate where Himmler had taken vacations with his wife from the early 1930s onwards. It was owned by an old party member and a family friend of Himmler’s, a veteran of the First World War’s Battle of Verdun—Hans B.^4^

## Introduction

This article focuses on giving voice to two members of the Lotta Svärd who were engaged in wartime nursing, both at the policy and practical levels, during Operation Barbarossa and until the final summer of the war in 1944. A close reading of two kinds of exemplary ego documents, one written by the Finnish chairperson and leader of a local Lotta Svärd district, Hilda B., and another written by her daughter, Elisa B., a voluntary military nurse—or as Lotta Svärd referred to them, Medical Lotta—reveals the ways in which Nazism exercised soft power in social policy and medical context. It delivers long-sought evidence^5^ regarding practical forms of cooperation and cross-border exchanges among Nazi women’s and girls’ organisations in the 1930s and 1940s beyond a single-country focus.^6^

Operation Barbarossa, the invasion of the Soviet Union by Germany and its allies was launched in the early morning on 22 June 1941.^7^ In this operation, Finland supported the Axis powers from the North through strategic pincer movements and provided Germany with significant army support, numbering nearly 500,000 men.^8^ Operation Barbarossa is referred to as the Continuation War in the Finnish context, as it followed the start of Finland’s involvement in World War II in 1939–40, when Finnish troops faced the Soviet Red Army during the anti-communist operation known as the Winter War.^9^

While there has traditionally been a manifest interest in the history of the central Lotta Svärd,^10^ Lotta Svärd’s regional authorities, which possessed significant amounts of autonomy and power and contributed to the functioning and international relations of the organisation—including Lotta Svärd’s relationship with Germany—have remained notably absent.^11^ Lotta Svärd had local chapters in every town, municipality and village; the organisation was particularly significant in rural areas, where a majority of the population lived. Structuring arguments from the regional archive in the Satakunta Museum in Pori, Finland—based on the collection of letters, speeches, diaries, statements, notebooks and other documents—this article argues that the nursing division of Lotta Svärd was given special attention by Nazi authorities and that Nazi social policies attracted greater interest in Lotta Svärd.

Drawing from recent studies on National Socialist social policy,^12^ this study examines both press and archival sources through two main dimensions of transnational Nazi social policy, as described by Kiran Klaus Patel. On the one hand, the function of Lotta Svärd can be considered as information exchange, in which a foreign model is used to legitimise existing or emerging national policies.^13^ On the other hand, as both partners made selective use of what the other had to offer, we are also dealing with selective policy adaptation, which builds on transnational exchange and learning processes.^14^ Here, it is suggested that Lotta Svärd represented an ideal women’s social programme to the Nazi regime. However, the nature of this exchange had a particular nuance—highly idealised and utopian—which was typical also for other states allied with or loyal to Germany at this phase of the war. From the early 1930s onwards, and increasingly during the later phase of Operation Barbarossa, beginning in mid-1942, both partners idealised each other. As evident in what follows, in fact, symbolic acts and reciprocal imaginings of the utopian other eventually proved more important than concrete acts of cooperation, which became increasingly uncoordinated, sporadic and futile.^15^ These visions were primarily promoted by the Nazi Party’s Office of Foreign Affairs, headed by the Nazi theorist and ideologue Alfred Rosenberg, and Rosenberg-related interest groups such as the Nordic Society (henceforth, *Nordische Gesellschaft*),^16^ a central mediation point for the German–Scandinavian relationship. In other words, while the *Reichsführer* Himmler considered Lotta Svärd a positive example and role model for the *SS-Helferinnenkorps* institution in 1943, the primary interest in Lotta Svärd was planted, and the character discovered by Alfred Rosenberg, already of interest a decade earlier.

The introduction begins with an analysis of the public image of Medical Lottas as it appeared in the main organ of the Lotta Svärd^17^ and examines how this compares with the National Socialist portrayal of the Lottas. Of the four main sub-sections of Lotta Svärd that were central to National Socialist image building was the nursing division, as it was considered the symbol of the organisation, and Medical Lottas were best considered to echo the ethos of the organisation.^18^ Moving beyond the frequently repeated assertion of the apolitical nature of nursing, the introduction illustrates that the image of the Medical Lotta became charged with political value. Depending on the needs of the German regime, National Socialism either appealed to traditional stereotypes or softened gender dualism when necessary.^19^ This mirrored an identical phenomenon at the time in Germany—a new era characterised by the higher status of and public esteem for nursing.^20^

The second part addresses the experiences of the two Lottas in order to gain insight regarding their actions within and reactions to the Third Reich. Hilda made a vacation as Lotta *Führerin* through Germany in 1944, as a guest of the SS organisation. In Germany she was received by SS, Third Reich medical officers and nursing organisations (German Red Cross, *Deutsche Rote Kreuz*; henceforth *DRK*) and women’s organisations (National Socialist Women’s League, henceforth *NS-Frauenschaft*). Her journey was the last of five known journeys made by the organisation to Germany^21^ in the late phase of the war and her travel diary is the only hitherto known testimony made by a leader cadre of Lotta Svärd in Germany.^22^ The diary reveals that beneath the realm of formal diplomacy, there existed a layer of overlapping private and semi-official social policy networks dedicated to cultivating bonds between the Lotta Svärd and its German counterparts.^23^ Instead, the account of Hilda’s daughter, Elisa, a part of the nursing community, examines a German medical unit in the operating room of a *Wehrmacht* hospital in Finland. It reflects the perspective of ‘accommodation’:^24^ the experience of an ordinary Lotta and her daily life in an exclusively male society. In honour of her medical care for Germans abroad, she was awarded the Order of Public Health by Adolf Hitler in 1943.^25^

To characterise the role of anti-communism in the overlapping utopian visions of Lotta Svard and the National Socialists, one central element is the Finnish Civil War in 1918. As with the Finnish Red Cross—whose medical staff had a politically conservative stance, and their leanings were with the Whites since the Civil War^26^—the origins of Lotta Svärd have an obvious attachment to the White cause and more broadly to the right-wing women’s movements across Europe.^27^ After gaining independence during the Russian Revolution in 1917, Finland entered into a civil war between the socialist Reds, backed by Bolshevik Russia, and the non-socialist Whites, backed by Imperial Germany. Indeed, Lotta Svärd’s popularity aligned, in particular, with those Finnish regions where fights between reds and whites had been the fiercest during the Civil War: these are Satakunta—the birthplace of Hilda and Elisa—and the so-called Finland Proper and Häme.^28^ In this regard, Hilda once remarked to the’White General’ Marshall Carl Gustaf Emil Mannerheim:^29^ ‘The people of Satakunta are rigid by nature. It takes a lot to get them excited or moved, but once it [Satakunta] warms up to a cause and takes it as its own, it carries it out with glowing enthusiasm—and when danger comes, it hits with a bear’s paws’.^30^

While Hilda herself openly linked her organisation to Reichsfrauenführerin, Gertrud Scholtz-Klink’s anti-Marxism, and to the stigmatisation of 1918,^31^ in the early years, the Finnish Civil Guard—the umbrella organisation of Lotta Svärd—had an intense interest in Italian Fascism as well.^32^ It established links with the Italian regime in the mid-1920s: Lotta Svärd’s earliest courtier can, in effect, be considered Benito Mussolini’s General, Alessandro Traditi, who profusely praised Lotta Svärd during his visit to the Civil Guard in 1926.^33^

As the archival material is highly personal and fragmentary, the transfer of medical knowledge cannot be established to the fullest. However, we know that, for example, the development of German modern medical science, child care, social work and the elevated position of mother and child were closely observed and selectively assimilated in Finnish nursing circles and Lotta Svärd.^34^ In particular, the *Mutter und Kind* programme affected the early development and consolidation of home-care work in Finland.^35^

Finally, this article builds on anthropological conceptions regarding nursing and medicine, thereby paying attention to culture-specific features of the given healthcare system, such as traditions, myths, hierarchies and cultures. In other words, I examine protagonists from their own perspectives, listen to their viewpoints, and bring to light a vision of their world.^36^

## The Prosperous Southwestern Idyll and the Origins of Lotta Svärd

Hilda, a long-time member of the Lotta Svärd’s Central Board, had a confidential and long-standing relationship with the chairperson and leader of Lotta Svärd, Fanni Luukkonen; the recipient of the star of the German Eagle Order, exceptionally awarded to a non-German woman by Hitler.^37^ Hilda’s position can, in fact, be compared to the *Gaufrauenschaft* leaders as she stood at the interface between the Lotta Svärd leadership and the lower organisational levels.^38^ Hilda, like Luukkonen, had the warmth and charisma of a somewhat older leader and, also like Luukkonen, was a persuasive communicator. Hilda belonged to the Civil War generation and had witnessed the imperial period and the Russian Revolution. And like many of the *Gaufrauenschaft* leaders had met Adolf Hitler, Hilda had met Mannerheim.

The depth of trust between the two can be perceived in some of Luukkonen’s letters to Hilda. In Luukkonen’s Lotta-idealisation, Hilda played a significant role. According to Luukkonen, Hilda epitomised Lotta: ‘In you, there is not a distinguishing scale between Lotta and a human. These qualities are harmoniously fused in you’.^39^ Luukkonen explained how ‘being Lotta, that is, yourself, you must amortise yourself’;^40^ she praised Hilda for having perfectly amortised herself and internalised the Central Board’s rules. For Luukkonen, Lotta emerged ‘at the intersection of the world of reality and imagination’,^41^ referring to the epic poem of Johan Ludvig Runeberg, *The Tales of Ensign Stål*, which includes a poem of Lotta Svärd following her husband to the Finnish War in 1808–09 to care for the soldiers and tend to the wounded.

By making no clear distinction between reality and fiction, Luukkonen’s ideal mirrored the antiquated concept of nursing, including its closely related element, myth, thereby embracing both the rational and irrational as well as credible and incredible elements.^42^ In contrast to modern functional nursing, in this older, need-oriented concept of nursing, ‘mysteriousness’ surrounded nurses’ activities, as they were still largely based on intuition rather than objectifiable observations.^43^ Luukkonen, whose writing was often tinted with a veil of romanticism, also acknowledged Hilda’s pivotal role as the founder and godmother of the second Lotta Svärd training centre, the ‘*Lottaheim*’ of Sorja, in the province of Satakunta in 1940: ‘Do you remember that late summer night when the rain hit the windows and we developed that idea [of Lottaheim]? When the morning dawned, the decision was ripe’.^44^

*Völkish* ideas and national socialist culture also revealed a distinct tendency to tend towards the irrational and emotional, closely resembling Romanticism.^45^ Proceeding from a mystical, romantic, even utopian point of view was highly characteristic of the national socialist worldview, particularly that of Alfred Rosenberg,^46^ not as much linked to mass propaganda but to underpin a suitable identity specific to the regime through concrete role models and heroic stories.^47^ In this sense, Rosenberg’s folkloric and ultimately religious–political considerations are a reminder of Luukkonen’s thinking. As evident in what follows, this ‘mysteriousness’^48^—through imagination and dramatisation—in wartime Lotta nursing became pivotal in the construction of the Lotta cult in Germany. Rosenberg himself highlighted the cultural, economic and historical ties between Finland and Germany: according to him, German and Finnish organisations and personalities had always kept the ‘old friendship’ alive; during the inter-war period, and particularly after 1933, the meetings of the *Nordische Gesellschaft* had renewed this friendship ‘in the most beautiful way’.^49^ In fact, Rosenberg’s ministry had a strong cultural policy orientation, which included the training of future Nordic elites in Germany and agitation against World Bolshevism. Illustrative of this is that from the moment Luukkonen first visited Scholtz-Klink in 1934,^50^ she maintained contact with Rosenberg at least until 1943, when Rosenberg, Scholtz-Klink and Luukkonen had dinner together at Scholtz-Klink’s guest house in Dahlem in Berlin.^51^ It may also not be a coincidence that the *Ahnenerbe* institute, whose patrons were both Rosenberg and Himmler, conducted research on Karelian folk healers, performers of traditional witchcraft, Finnish national epic Kalevala, pre-Christian religion, fairy tales and magic in the North Karelia and Savolax regions at the same time when Lotta Svärd’s public discourse elevated the women of Kalevala as ancient models of Lotta Svärd.^52^

With regard to the construction of the public image of Lotta Svärd, many of Hilda’s speeches highlight her own southwestern distinctiveness and hints of exceptionalism and stress how essential this region is—with its persisting senses of traditionalism and conservatism—for an adequate understanding of the Lotta Svärd culture and identity. Hilda designated the traditional role of leisured middle-class ladies dispensing bounty to the deserving in this idyllic and prosperous southwestern village environment: ‘The first Lotta Svärd organisations were established in the vast expanses of the cultivated fields of Satakunta already in 1919, with funds of patriotic sewing circles established by ladies of houses’,^53^ that is, wives of planters and landowners of the region. Consistent with this romanticised image of the South, Hilda also mentioned the work of American women during the US Civil War of 1861–65 as comparable to the ‘tenacity and determination with which women worked during the Civil War in Finland’.^54^ While sounding much like a conventional description of American antebellum society,^55^ Hilda highlighted the decisive connection between (her own) southwestern identity and the origins of Lotta Svärd: the belief in women who grow over time into people of remarkable courage and resiliency and a belief in the sanctity of the home. By mentioning American women and picturing Lottas as ‘lion-hearted and self-sacrificing, like the women in ancient Sparta’,^56^ Hilda emphasised nursing work’s sacrificial nature, an attitude marked by humility and self-denial,^57^ and the fact that nursing is considered an act of Christian charity. In fact, a recurrent trait both in Lotta Svärd’s rhetoric and that of German women’s groups in the 1930s–40s was the attitude of avoiding excessive sensationalism.^58^

As evident in what follows, this originally southwestern, genteel and pious model of Medical Lottas—as conceptualised by Hilda and promoted by Luukkonen—simultaneously took control of the media and culture in Finland and Germany. This rise of Lotta’s public image occurred not only concurrently with Lotta Svärd’s intensifying connections to the ‘new Germany’ but also on the threshold of National Socialism and—particularly strikingly—when a new era in German nursing, characterised by higher status and public esteem of nursing, began.^59^ During this time, on the one hand, German Red Cross officials charged nursing with a new, political value to improve the profession’s social status; on the other hand, they made it possible for a profession with an ‘apolitical consciousness’ to be subsumed as a part of the larger political system.^60^ In fact, playing down the political nature of organised women’s activities was a characteristic feature not only of the German Red Cross and the *NS-Frauenschaft*^61^ but clearly also of Lotta Svärd.

From the earliest mentions in the German press,^62^ Medical Lottas were elevated to a position of public respect. However, it was really only after Fanni Luukkonen had met Rosenberg at the ambassador’s tea party in 1934^63^ that the construction of a proper Lotta cult began in Germany. Immediately after Luukkonen’s visit, as if echoing Rosenberg’s picture of the North, which specifically included Finland,^64^ the ‘*Reich* Romantic’ portrayal of Lotta—using fairy-tale style narrative and ideals of Nordicism^65^—appeared in a regional German newspaper. Creating a link between a cultural theme and contemporary political development, the newspaper’s ‘Story of a Strange Encounter’ introduced a chaste and patriotic Lotta as an object of imagination and a focus of national fantasy.^66^ In the story, a German pilot takes off from Germany to chase the mysterious plane *X*. He ends up in Lapland and suddenly meets the mythic Lotta and her pilot husband, whose aircraft carried a symbol similar to the national socialist party emblem, which was used by the Finnish air forces at the time. Sitting in the hangar around a humming samovar, the German pilot contemplates the slender Lotta with blonde hair, her ‘blue and clear’ eyes in particular, and her movements, which are ‘calm and serenely beautiful’.^67^ Yet the German press offered also a completely opposite image—that of a masculinised and heroic Lotta. Likening Lotta Svärd to the ancient Germanic women of the Teutons, who defended the Wagon Fort with men and naming Lottas as Amazon corps,^68^ was obviously indicative not only of Rosenberg’s neo-medieval and chivalric dreaming but also of the military deployment of women.^69^ To summarise, as if echoing Hilda’s speeches, Lottas’ public image followed a dual picture: depending on the desires of both regimes, it emphasised contrasting notions of femininity—that is, Hilda’s southwestern ideal (internal and external ‘Nordic’ qualities) or her hardened or masculinised femininity (the ‘Spartan’ qualities).

Similarly, the *Lotta Svärd* journal became an ideal format in which pre-existing conceptions of nursing—such as femininity, physical fitness, discipline and militarism—were altered to cater to larger political uses.^70^ One editorial in the journal even acknowledged that the journal’s ideas, for a long time, had been ‘guided by the Rome–Berlin axis’.^71^ Just as in the German ‘Story of a Strange Encounter’, one recurring characteristic was the use of sharpened gender divisions, specifically in representations where encounters between German officers and ‘ordinary’ Medical Lottas were portrayed. For example, when General Nikolaus von Falkenberg reviewed a German war hospital in Finland in 1941, a Medical Lotta depicted the General as a pleasant man who valued Medical Lottas’ work highly and treated them ‘like any precious woman’.^72^ A recurrent feature in such representations is that the German officer is never seen as isolated but always surrounded by truly feminine women, present in men’s own self-image to support military masculinity.^73^ The Medical Lotta calls von Falkenhorst great ‘lord’ (*Herr*), while the general calls the Medical Lotta ‘*Die Kleine*’ (the little one), as she was his ‘old friend’.^74^ Similarly, when Fanni Luukkonen reviewed the Lotta Svärd guesthouse on the white-sand beaches of Yyteri (Pori) in 1943 with the Hungarian ambassador and minister, the ambassador’s heart was deeply touched by ‘*die blonde Kleine*’ (the little blond).^75^ As if echoing German portrayals of Medical Lottas, Luukkonen personally promoted an ideal of masculinity elevated to the heights, fulfilled by appropriate femininity to the German troops through different channels.^76^

As the examples outlined above illuminate, one powerful method of National Socialist communication was to appeal to traditional gender stereotypes and to gender complementarity.^77^ But while the rigid separation between male and female spheres was important, in political and social reality, Nazi rhetoric always softened this dualism when it appeared necessary. As shown in the following sections, this softening led to what Steinbacher calls a flexible fighting community and militarised comradeship of men and women.^78^ In fact, Lottas’ public image transformed into a kind of philosophical masque in which everything stood for a certain vast (political) idea.^79^ Such representations were rather close to the heart of fascism and its politics of aesthetics.^80^

## The Dream of Mother ~ Lotta *Führerin* in Germany

Hilda’s journey through Germany in the summer of 1944 represents the interaction and exchange between an Axis ally and the Nazi regime during that phase of the war which was characterised by a growing physical separation. Yet, as Hilda’s diary reveals this growing separation did not imply that the issue of social policy disappeared. In contrast, there was a particularly warm reception for sympathetic regimes. As we shall see, symbolic acts and reciprocal imaginings of the utopian other eventually proved more important than concrete acts of cooperation.

Hilda’s diary is a contemporaneous source in which she reports on her experiences in a private, rather than official, capacity. Pragmatic in approach and using simple words, the diary is written in a brief, terse style, which is suggestive of a writer with little time for diary-keeping. The writing may even have been completely forbidden, as strict censorship was imposed. Although she avoids using any names in the diary, when reading it simultaneously with her guestbook,^81^ it is possible to connect times and places, and, in a few cases, names. Furthermore, a few destinations are openly mentioned, such as *SS-Hauptamt* in Berlin or *NS-Frauenschaft* and the *DRK*.

Like *Gaufrauenschaft* leaders, in Germany, Hilda carried out representative tasks and represented the organisation and its chairman.^82^ She participated in numerous *SS-Hauptamt* and *NS-Frauenschaft* comradeship evenings, the first of which she attended soon after arriving at the *Reserve-Lazarett* VIII military hospital in Stettin on the last day of May 1944.^83^ She was received by an unnamed staff surgeon, a *Wehrmacht* medical corps officer with the rank of captain and a matron from the *DRK*. From 1933 onwards, *DRK* provided support for the official medical service of the NS regime and demonstrated acceptance of National Socialism.^84^ After a day-long programme, which included a variety show with cabaret—dance, and magic tricks—the local SS leadership organised a welcome reception in the hospital premises adorned with swastikas and garlands and a military orchestra. The local SS leader wished the Lottas ‘rest and health’, to which Hilda responded by handing the *Stabsarzt* a knife, medical literature, and Runeberg’s work *The Tales of Ensign Stål*.^85^ By handing over these gifts, Hilda emphasised the equal importance of the knife to the activities of a surgeon as to the activities of a soldier; the knife was for ‘work, eating, and fighting’.^86^ As gifts with symbolic meanings were particularly fostered by the SS system, Hilda appears to have been aware of the symbolic method of communication—imprecise and mythical in nature—to get her message across.^87^ Evoking a connection between the nursing and military disciplines, a month later she donated another symbolic knife in Salzburg to another *Oberstabsarzt* and *Sturmbannführer*, a medical corps officer with the rank of major who as a rule commanded field, evacuation or war hospitals.^88^

After arriving in Berlin on 1 June 1944, the Lottas were scheduled to have afternoon tea with Himmler in Tiergarten, in the *Nordische Gesellschaft* office.^89^ Although Hilda remained imprecise regarding the location, she noted that they entered ‘through ruins to a magnificent apartment’. Himmler’s schedule had changed and, eventually, the admirer met his objects of admiration that same evening at a dinner in Potsdam. After the *Nordische Gesellschaft* tea reception, there is very little information given about the dinner other than Hilda’s terse note of thanks, which was marked in her notebook as ‘An den Reichsführer SS. Heinrich Himmler’.^90^ The only references to the events of the evening in Regattahaus are Hilda’s description of the excellent food, fine performances (by the first tenor of the German state opera and a Finnish violinist), and speeches given by Hilda and a Finnish SS officer.

When she arrived in south-eastern Bavaria, at the estate of the NSDAP party veteran and SS district leader Hans B., Hilda was kept, in her own words, ‘like a bishop in a vicarage’.^91^ She mentions relaxation and ‘conifer and full pine baths’; long hikes through the mountains; and, at night, when tired, falling asleep under Hans’s down feathers.^92^ In Bavaria, Hilda’s writing is heavily influenced by time and place. Overall, her description of the Alpine, Bavarian and Austrian nature illustrates her idealization of the Nazi Germany as a social utopia. She evokes idyllic scenes and private bucolic moments, capturing even the sounds and smells of nature. Walking in the Hochgern Mountains among anemones, gentians and spring primroses with a shepherd and his hundreds of sheep, who followed him perfectly, Hilda evokes the universal will and active force in nature and senses the ‘nearness of God’: ‘We felt our smallness and the greatness of God’.^93^ This style of thought attracted national socialists, for whom the will immanent in nature was the functional equivalent of God.^94^ After having goat’s milk, real coffee and bread with marmalade in an alpine hut, she returned to Hans’s estate that evening; Hans gave her a copy of Hitler’s *Mein Kampf*. Under the circumstances, the final year of the war and the iconic setting intended as spiritual ‘re-fuelling’ feature prominently in Nazi aesthetics and acquire distinctly political undertones. The aesthetic appreciation of the Alps, as described by Hilda, takes on political gravitas.^95^

However, reciprocal imaginings and visions of the utopian other did not always have connections to actual conditions. Martha from the *NS-Frauenschaft,* who had learned to ‘know and love *Lotta Svärd*’ at the day of the beginning of the invasion of Normandy on 6 June 1944 in the Bavarian mountains, projected an unreal and unworldly atmosphere.^96^ She daydreamed of sitting with Hilda on their beloved bench high in the mountains under the gorgeous sun and sensed that Hilda might still have a connection with her and be thinking of her. An interesting detail is that Martha called Lotta Svärd a sect: ‘May God always protect you and give you power and strength for your sect’.^97^ Subsequently, Martha sketched a future that never materialised. She proposed information-sharing between Lotta Svärd and an unnamed entity for the future ‘through a kind of newsletter…’^98^. Lotta Svärd would have been welcomed in Berlin and cherished there in the ‘next few years’.^99^ In intelligence terms, Martha may, in fact, have been a ‘tail’ of Hilda, yet we do not know how Hilda responded.

At Hans’s estate, Hilda received numerous visitors, such as Traunstein’s SS-Colonel or the head of the local *NS-Frauenschaft* who arranged an evening in Hilda’s honour that could be understood as pseudo-participation in an NSDAP local evening. Hans’s entry from this evening in Hilda’s guestbook reflects his explicit deification of both Hitler and Mannerheim as the ‘only hope for Germany’s salvation’.^100^ Hilda gave a speech on the history and function of Lotta Svärd, and *Deutschlandlied* and *Horst-Wessel-Lied* (the anthem of the NSDAP) were sung.^101^ On the day of the solstice, *Jugend* songs were performed in Hilda’s honour by the *Jugendgruppe* of the *NS-Frauenschaft*.^102^ At the estate, Hilda was introduced to the medical beliefs of ‘*Neue Deutsche Heilkunde*’, promoted in the Party doctrine, particularly regarding nutrition, medicinal herbs and bathing; the deputy leader of the local *NS-Frauenschaft* lectured Hilda on nutrition, using living plants as medicinal botanicals and nutrients directly from nature in times of need,^103^ and the chief physician of Traunstein’s SS hospital and convalescent home for SS members and their families introduced Hilda to a variety of holistic treatments for military patients, such as massages, gymnastics and a variety of baths.^104^ This facility was functioning under the association of the *Erholungsheime für naturgemäße Heil- und Lebensweise e.V.*, originally instigated by Himmler.^105^ The speciality in this hospital was a Finnish sauna. In fact, sauna bathing for preventive and therapeutic means was among Himmler’s topics of medical research and trials in 1943. Himmler also promoted the German Sauna Society.^106^

Many of the narratives left by nursing staff such as *DRK Schwester*s, *Helferin*s or *Leiterin*s from *Reichsfrauenführung* in Hilda’s guestbook celebrate the relationships between the *DRK* and Svärd and between the *NS-Frauenschaft* and Svärd.^107^ Many of these are amateur poems and philosophical quotations that contain explicit and implicit political content and language. In an attempt to encourage enthusiasm for the political alliance, the three ‘Berliner *DRK* Helferins’ greet Hilda with comradely solidarity and express the irrepressible pride of being able to shape the European ‘resurrection’ while serving the *Volk.*^108^ Much in the same style that the *Reichsführer* Himmler considered Lotta Svärd a role model for the SS-*Helferinnenkorps* institution—something that an ordinary German woman should look up to and take as a guide^109^—Vera S. refers to the ‘dearest little Mama, who will always be a role model for us’.^110^ Attaching ideals of quiet selflessness and commitment to Hilda, Vera also quotes an author from German Romanticism, Friedrich Hölderlin: ‘*Was wir sind ist nichts, Was wir tun ist alles*’ (‘What we are is nothing. What we do is everything’).^111^ Gisela T. calls Hilda a female messenger from Finland and partially quotes Goethe’s poem, often called ‘*Feiger Gedanken*’, which implies masculine bravery, ‘Germanness’, the strength to defy all powers, in order to call the gods to one’s side in contrast to the ‘cowardly thoughts of anxious vacillations’ and ‘womanly wavering’.^112^ Another entry by Vera S. features Nietzsche’s poem ‘*Heraklitismus*’, which refers to the essence of friendship and the rarity of brotherhood, ending with the following ominous words: ‘equality’ before the enemy and ‘freedom before death’.^113^ Strongly present is either the ‘Spartan’ or the ‘Nordic’ ideal: the chaste ‘white nurse’ who appeared either as a mother or a sister figure—the one who would be at a man’s side as a comrade.^114^ Instead, Thea S. wrote a proper panegyric to Mama-Hilda and to the quiet beauty of a country in the North and ended her dedication with a welcome to Lotta Svärd to Germany.^115^ These narratives, referring to Lotta Svärd as a role model and a heroic story obviously reflected the indoctrination principles of the regime, yet they also represent dream projecting and utopianism, highly central to Nazi ideology.

Many of the entries left by nursing staff display another recurring vision that may be categorised as utopian: the religious element. This element appears either in the form of prayers, asking for blessings for her *patria* in fateful days or turning everything over to divine guidance.^116^ Many of these dedications elevate the figure of mothers in a holy position—an obvious indication not only of the centrality of motherhood in 1930s Nazi German ideology but also of the nurse’s role as a gendered symbol of domestic relationships, such as familial or maternal figures.^117^ In these, Hilda is often called either ‘*Mutter*’, ‘*Mama*’ or ‘*Oma*’ (Granny). In fact, the extensive use of terms and elements of the church/Christian liturgy was not uncommon in the rhetoric and language of national socialist women’s organisations.^118^ Christianity, for Nazis, was a highly ambiguous concept, neither openly rejected nor encouraged.^119^ For example, with her Christian-religious choice of words, Scholtz-Klink’s public speeches often emphasised the compatibility of the Church and National Socialism, but in Hilda’s guestbook dedications, there appears a truly specific trait—the attribution of divinity to women.^120^ A renowned Christmas carol was apparently performed in Hilda’s honour during one *NS-Frauenschaft* evening. The lyrics of this carol were changed and, thus, the protagonist of the song, the Christ Child, is substituted by a mother, who, like Christ, is coming soon. The original chorus, ‘Freue Dich, Christkind kommt bald’, was changed to ‘Freue Dich, Mama kommt bald’.^121^ Indeed, as Christiane Berger contends—in the rhetoric of national socialist women’s organisations—it was not unusual to attribute divinity to women or elevate women (for their ‘creative power’) to the position of God. This is exactly what happened with the ‘arrival’ of the Lotta Svärd’s district leader in Germany.^122^ On the whole, Hilda’s diary reveals that even under heavy Nazification, the Christian nursing traditions survived among the members of German women’s associations and *DRK Schwester*s. They included not only forms of devotion, such as songs and prayers, but also non-verbal religious practices.^123^ Although National Socialism usually had an anti-clerical orientation, for example, in the assessment of political and ideological reliability of *Helferin*s, belonging to a church or religious community was considered an indicator of a person’s political reliability and even of a national socialist worldview.^124^

This connection between warfare and religion, as well as the utopian and euphoric component in Hilda’s diary entries, may be explained by the fact that Bolshevism was considered anti-Christian ideology. This echoed strongly in the nursing and medical spheres in Finland. Nursing historian Virtanen indicates the specific context of the Continuation War, which was considered to have a religious–ideological nature; Mannerheim and a few Finnish media considered it as a ‘holy war’.^125^

Approximately 2 weeks before her departure from Germany, Hilda’s diary ends; however, her guestbook reveals that after leaving Bavaria she visited the SS main office, which was based in Douglasstrasse 7-11 in the Grünewald district of Berlin,^126^ which was also the last liaison office of Finland, *Finnische Verbindungsstelle*. During her last week in Germany, Hilda moved to the area of contemporary Poland; there, in Gdánsk, she visited the facilities of *NS-Frauenschaft* and the *DRK*. In Sopot, she met with the DRK’s *Oberstführerin*, a female *DRK* rank, roughly comparable to a colonel.^127^ She also visited the *Führerschule* in Gdynia, where there was an immigration centre which oversaw the NS resettlement programme and deportation of the Poles,^128^ and she also met with the *Hauptführerin* of the *DRK Führerschule*.^129^

## ‘Sanitäts-Lotta’

As shown in the previous section, while Lotta Svärd’s international relations nurtured an ascending relationship with Nazi Germany throughout the 1930s, a practical relationship with German medical staff intensified during Operation Barbarossa. There was a strong presence of the German army in Northern Finland. As part of the intra-Axis social policy cooperation, soon after the outbreak of the war, the German medical administration turned to the Medical Department of the General Headquarters of Finland to recruit nurses and Medical Lottas.^130^ Even if the tightest relationship with the German army occurred in professional spheres, such as in the medical section, written individual accounts or diaries focusing on wartime nursing in German-led war hospitals in Finland are very rare.^131^ Hilda’s daughter, Elisa, delivered a 19-page presentation regarding her experiences and daily work in the operating room of a German war hospital in Northern Finland to a closed audience—her dentist colleagues.^132^ By exploring her account, it is possible to highlight the transnational character of intra-Axis voluntarism.^133^

Compared to her mother’s diary, the language and style Elisa adopts in her account bear witness to her educated background. Factual details stand alongside more reflective passages. After all, Elisa was from a wealthy background, with a high-ranking Lotta Svärd district leader as her mother and the chief physician of a local hospital district as her father. In summary, she was a trained doctor (a dentist) herself and, thus, a woman in medicine. With the self-representation of an ambitious woman doctor, she distinguished herself from lower-skilled assistants, even from the work of other Medical Lottas. Elisa’s writing can, indeed, be paralleled to the writing of voluntary nurses, as studied by Christine E. Hallett.^134^ When comparing texts produced by trained professional nurses and voluntary nurses, we can see that those produced by the latter are often more reflective, analytical and occasionally assertively opinionated, which is true of Elisa’s writings, while the former tend to be factual and pragmatic.^135^

Although she reveals that she spent 2 years at the hospital, beginning in June 1941,^136^ Elisa is rather circumspect regarding what she conveys to the public; she does not report any factual information regarding the location of the hospital or disclose the identities of those mentioned in her account. Nonetheless, based on entries written in her guestbook by German patients,^137^ the hospital can be identified as *Kriegslazarett* 1/521, a German military medical unit in the active theatre of war in Northern Finland, which was part of a newly built modern hospital in Kemi. The unit was a stationary/base hospital set up in a vacated facility, a local school. It offered 24-hour coverage for a permanent 400-bed hospital that was divided into wards of 30–110 beds for wounded and sick German soldiers.^138^ The unit housed short-term patients who required immediate post-operative care or stabilisation in preparation for evacuation to Germany or Norway. As the evacuation distances were long, the casualties were transported by train, car or aeroplane to this unit for further surgical care. The patient turnover was rapid; a ‘typical’ patient would recover for approximately 5 days before being evacuated. In this unit, there were three to five German physicians and *Schwester*s and a Finnish principal matron, who acted as a liaison between the Germans and the local administration. Every ward had a ward nurse, one or two nurses, and a variable number of Medical Lottas, depending on the size of the department and the condition of the patients. The hospital operated on German time.^139^

Although originally signed up for a post that corresponded with her civil profession, dentistry, Elisa was assigned to become a Medical Lotta. Only after one and a half months of having the status of a lowly probationer as an orderly, was she finally transferred to the operating room as a Medical Lotta, primarily serving hospitalised male patients. Although Medical Lottas worked as nursing assistants under the supervision of trained nurses,^140^ Elisa emphasised that her work was similar to that of an operating room nurse. She worked closely with a physician, surgeon and anaesthetist; assisted in the surgery; administered narcotics; dressed and plastered patients’ wounds and cleaned up after surgeries.^141^

With regard to the German medical and nursing culture, Elisa made observations that were, on one hand, based on distinctive individual behaviours; however, on the other hand, commenting on modes of organisation. With regard to the overall organisation of the hospital, Elisa noted that it was created to be ‘as practical as possible’; she summed up that ‘everything was organised with resources characteristic of a great power and with German thoroughness’.^142^ The unit was a highly disciplined environment in which obedience and compliance were appreciated abilities. The German physicians provided top-class medical care and had advanced, even lavish, financial resources at their disposal; moreover, they were well-supplied with instruments, equipment and pharmaceutical products, including Elisa’s own specialisation, dentistry, where patients had ‘all possible treatment’ available at hand.^143^ Although the work in the operating room was extremely exhausting and shifts often lasted until the middle of the night, the working hours, environment and duties were prescheduled and less chaotic than, for example, that of field hospitals.^144^

The operating room, situated on the top (fourth) floor, was adapted from a large drawing room of the former school, which was divided into two by a partition wall. There were three operating tables as well as one German *Oberschwester*, two German *Schwesters*, two German *Sanitär*s, two medical scribes, three Finnish nurses and Elisa as Medical Lotta worked there continually. As all dirty, festering wounds and amputations were treated in this same room, no surgeries that required absolute sterility could be performed here. Instead, countless removals of shrapnel and hand and foot amputations were performed and *tourniquets* were applied.^145^ In regard to transfusions, Germans never used bottled blood, relying instead on the more antiquated method of transfusion made via syringe directly from a donor of the compatible blood type to the recipient.^146^

The hospital became a forum for scientific and professional exchange and a vantage point for observing German scientific excellence. Elisa reports on numerous procedures of interest, such as a very ‘carefully performed treatment of bone fractures’,^147^ likely linked to Professor Gerhard Küntscher, who was the chief physician of *Kriegslazarett* between 1942 and 1944. Küntscher introduced the Küntscher nail,^148^ an intra-medullar nailing of bone fractures, a novel technique that enabled German soldiers to make considerably faster recoveries than patients treated using other means. During his posting, through his teaching of local surgeons, Küntscher set a broad example for the German training system in Finland. For example, in 1943, he gave a lecture and performed two surgical demonstration operations during the annual meeting of the Finnish Society of Surgery in Helsinki.^149^ When the *Kriegslazarett* was evacuated in the fall of 1944, Küntscher, who left on short notice by helicopter, left many of his nailing instruments to the hospital’s chief military surgeon.^150^

Elisa experienced a certain degree of conflict with the notion of her education, gender and femininity in a masculinised environment. With regard to the level of perceived professionalism and status, although the treatment Elisa received from the German medical staff was ‘impeccable’, the German doctors and *Schwester*s had misconceptions regarding the knowledge, skills and status of both Lottas and trained nurses: ‘German doctors performed many of the tasks that a trained nurse would perform in our hospitals’.^151^ Both physicians and patients were conscious of her lower status, particularly at the outset. Because of such preconceived ideas, ‘Lottas were expected only to be and do, but not to be seen’.^152^ This appears to be linked to an order given by the German medical administration, according to which, during the doctors’ ward rounds, Lottas were not allowed to be seen, unless indispensable, such as when acting as an interpreter. However, there were also tensions between the Finnish nurses and Medical Lottas in her unit.^153^ As discussed in the previous section, as volunteer nurses, Medical Lottas were ‘celebrities’ who were more prominent in the eyes of the general public as compared to their trained supervisors who worked more in the shadows. This notoriety, and the presence of Medical Lottas in the nurses’ domain of practice, clearly caused tension between individuals and between organisations.^154^ The women who nursed voluntarily, rather than professionally, captured the imagination of the reading public and were considered romantic figures.^155^

Whereas the recruitment to the German medical service implied granting women their own realms of comradeship, that is, extending the frontline community to include women, Elisa’s account implies that this ought to be done without questioning the superior role of men. In fact, despite all the expansion of female responsibilities during the war, gender differences remained fundamental in German militarised society.^156^ This becomes particularly evident in Elisa’s description regarding nurse–patient relationships. On one hand, Medical Lottas stayed very near the patients, often acting as adepts to patients’ personal matters or as their confidants;^157^ in contrast, trained nurses maintained a certain emotional distance from the patients. During the ward rounds of German doctors, which were grandiose, Medical Lottas were often questioned about the soldiers’ social and private spheres and served as interpreters between the patients and the doctors. Medical scribes made notes on their papers, and based on this information, carriers picked up patients on stretchers for examination in the operating room. Apparently linked to medical confidentiality, Elisa drew the following conclusion: ‘Hence, besides artery and temperature, nothing was really known about the patient in the ward by the nursing staff’, except for physicians and the Medical Lotta (who knew everything).^158^

Medical Lottas also assisted the patients in rites of passage, such as their transition from an ill person to a healthy one, and in their discharge and leaving for the front. Here, it must be emphasised that the issues that nurses had to console German soldiers about were unique to the context of the war on the Eastern Front, where the men had both witnessed and participated in mass violence. Such acts, particularly in Russia, had serious psychological effects on the soldiers.^159^ Presumably, Elisa also learned first-hand how the men coped with regular encounters with, and participation in, mass death. Nevertheless, she exhibited no dislike for wartime German brutality, but instead showed a rather caring attitude towards her German patients.

On the other hand, Elisa’s narration displays a strong, enlisted men–officer dichotomy. For example, she brings out ‘the great difference that existed between the genteel sophistication and finesse of German officers and the enlisted soldiers’.^160^ As professional hierarchies were rigidly drawn in this hospital, patients were classified into two groups that ‘differed sharply from each other in every respect’: officers and soldiers.^161^ These two categories, according to the ‘German concept’, were ‘as far apart from each other as a button is from a star’.^162^ Similar to how Prussian officer-patients were described a hundred years ago as ‘men of fine physique and manly bearing’, ‘of cool undaunted courage’ and admirable in the ‘discipline under which they have been brought to such perfection’,^163^ Elisa portrays the high-ranking German officer-patients as arrogant, lordly and high and mighty.^164^ Even when assuming the role of patients, German officers exuded authority, self-confidence and were revered by the medical staff. The officer-patients were kept in separate rooms, and, in Elisa’s words, ‘were literally worshipped’.^165^ Implying their lack of gentleness, Elisa laments that the highest-ranking officer-patients felt above all the regulations and restrictions of the hospital and assumed the right to order and commanded Lottas ‘like their own men’.^166^ She provides an illustrative example of when principal matrons invited the entire staff of the operating room for a coffee, including the ‘gentlemen doctors’, who held the rank of officer—a gesture that was not appreciated by the officer-patients. The officers left very quickly and subsequently explained to the matron that they would ‘never sit in the same room with a soldier’.^167^

To summarise, in Elisa’s narration, one can find the typical dichotomy, wherein the officers are described as the ‘worst ones’, while the enlisted men are the good ones.^168^ Whereas the image of traditional masculinity in Elisa’s account is embodied by the officer cadre, the masculine ideal of the enlisted soldiers also integrates more feminine manliness, tenderness, caring and soft sides. Wounded German men are described as beautiful boys with gentlemen’s behaviour who never swore or talked dirty. Both officers and soldiers were highly devoted to neatness, cleanliness and their appearance. Elisa conveys sensations of aesthetic pleasure in seeing them ‘impeccably shaved and properly washed’. Her male patients were all ‘well-groomed, even dandified, being as beautiful as bijous’, they ‘smelled pleasant, used scented lotions and scented toilet waters,’^169^ all of which may reflect a national socialist focus on cleanliness and how it may have impacted the thinking of nursing staff. A maternal style is particularly used when describing the enlisted soldiers, who were kind, polite, helpful and, in their minds, ‘as sensitive as children’.^170^ As seen above, in spite of sharply drawn gender differences endorsed by the German army as an institution, role models for ‘battlefield men’, particularly during the later stage of the war, were made more flexible.^171^ The concept of comradeship in the ‘total war’, in which traditional moral considerations had been eliminated, allowed the display of tenderness and toughness in one person.^172^

In January 1943, Elisa was awarded the Reich’s social welfare decoration, ‘*Medaille für deutsche Volkspflege*’, signed by the *Führer* and his head of the Presidential Chancellery, Dr Otto Meissner.^173^ This medal was generally awarded to honour members of the Reich and foreigners. The proposals for the award were submitted to the relevant body, which then submitted the corresponding proposals to Hitler, who ultimately decided on them himself.

## Conclusion

In late July 1945, Fanni Luukkonen wrote to Hilda. She was in *Lottaheim* Sorja and afraid to ‘meet memories’ by returning to a place so dear to both of them, to their ‘shared autumn night dream’.^174^ Luukkonen implies the good old days but also considers the wider political narrative that framed the two leaders’ experiences. She expressed nostalgia not only for the lost time but also for the loss of the political frame. Luukkonen found herself isolated from the previous world and felt she no longer had a place in either after the armistice. She appears to have doubted her identity and questioned her ability to fit back into the civilian world. She wished God’s blessings for Hilda and herself, the ‘little wanderers’, whose direction was now towards the ‘western evening’.^175^

This study addressed two main themes. First, we have seen how Medical Lottas’ public image in the German press began to grow simultaneously with Lotta Svärd’s intensifying connections to ‘new Germany’, and specifically after Fanni Luukkonen met Alfred Rosenberg, the most important cultural–political ideologue of the Reich, in 1934. Already the inter-war period witnessed intensifying forms of exchange between German state actors and experts on a social policy programme like Lotta Svärd. This period signalled a new era in German nursing, characterised by a strong relationship between health and politics as well as by the heightened status of and esteem ascribed to nursing. On the whole, as Thomas Kühne notes, the Third Reich pushed women more deliberately to the public sphere than any other political regime in Germany had done before.^176^

Second, the archival materials written by Hilda and Elisa provide insight into how Lotta Svärd maintained connections with Germany at the grassroots level. While Elisa’s account reveals the social reality of intra-Axis transnational social and labour policy cooperation from the viewpoint of an ordinary Lotta, Hilda’s account reflects Lotta Svärd’s institutional high-profile relations with the Third Reich. Lotta Svärd was received by comparatively high-ranking representatives of the SS organisation, medical units headed by Nazi medical officers, nursing organisations (e.g. the *DRK*) and women’s organisation (*NS-Frauenschaft*). Lotta Svärd’s contacts with the Third Reich were managed largely via middle management—that is, through district leaders, the equivalent of German *Gaufrauenschaft* leaders. In fact, Hilda was mainly received by the heads of the NS-*Gaufrauenschaft* and occasionally by local *Gauleiter*s.^177^

Regarding the Nazi social policy, the elevation of Lotta Svärd’s public status in Germany, the praising tone of the Third Reich regarding Lotta Svärd and the long-standing positive commentary on Germany’s social policies and scientific excellence in the official journal of Lotta Svärd represent that information exchange in which a foreign model is used to legitimise existing or emerging national policies.^178^ As the journal *Lotta Svärd* featured articles praising Germany’s military and social achievements, such as women’s associations, and contrasted them with anti-Bolshevist ideas, *Lotta Svärd* became a tool for enhancing Germany’s international influence and the pursuit of its foreign policy goals. The mutual praise of Lotta Svärd and German leaders for each other’s policies was due to efforts to quell communism and fears of it.^179^ This entanglement of science and symbolism is much in line with Konrad Jarausch’s concept of ‘organic modernity’; an alternative, utopian version of modernity which aimed to strengthen the nation against two other modernities—the liberal or socialist rivals.^180^

Instead, the selective policy adaptation noted by Patel was exposed by Luukkonen herself. In 1934 she contended that her impressions from Germany would enrich her organisational work: ‘It is good to keep what is good’ in the German model,^181^ thereby inferring that it was necessary to draw inspiration from the solutions adopted in Germany without necessarily adopting all the methods or subscribing to all the values promoted by Nazis. This is a typical selective policy adaptation, building on transnational exchange and learning processes.^182^

However, as Patel contends, the international references and contacts by Nazi leadership were not merely meant to propagate a distinct form of female fascist networking. They may also be seen as part of the power struggles between competing factions of the Nazi state.^183^ In fact, it is possible to suggest that Rosenberg and his ministry understood the cultural policy value of the Lotta Svärd very early on. While Lotta Svärd was apparently close also to the *Reichsführer* Himmler, as he declared Lotta Svärd as a role model for *Helferinnenkorps*, we argue that it was Alfred Rosenberg who discovered the character of Lotta Svärd in the first place. As Michael H. Kater contends, both men shared the same historical and cultural views, which made the two rivals.^184^ Additionally, it can be argued that the heightened mutual interest in each other’s social policies between 1933 and 1945 was connected to the plans of Greater Germany (*Großdeutschland*) that would include Scandinavia^185^ and in which Lotta Svärd had a role to play.

Hilda’s and Elisa’s accounts were probably never intended to reach a wide audience. Both wrote about their experiences for personal reasons, but they never shared them with the world. Hilda had a significant understanding of politics and she likely saw herself as a political actor, while Elisa did not clearly convey political themes. In line with her position as a district leader, Hilda’s writings provide clear evidence of her patriotism and enthusiasm regarding the ‘new Germany’. Regardless of the seriousness of the subject matter, neither dealt with difficult topics or moral dilemmas. There are no comments on humanitarian matters or on the larger context of the war. Yet, because of the nature of their work and their closeness to German officers and soldiers, both contributed to the *Wehrmacht* government’s efforts.

The Spartanly simple, bureaucratically conscientious, and morally ‘exemplar’ organisation in which nursing assistants from humble origins served as the protagonists had primarily a symbolic meaning for the national socialist regime. Yet, behind the praising tone used by the Third Reich for Lotta Svärd, there was most likely something more to it than simple propaganda or public image building. Building on the work of Clifford Geertz on cultural systems,^186^ Lotta Svärd echoes the sacred place the ‘*Mutter*’ held in German society, thereby signalling the centrality of motherhood in the national socialist ideology of the 1930s as well as that of the nurse’s role as a gendered symbol of domestic relationships. Lotta Svärd offered a suitable cultural frame in which political authority and the office of the leader may define itself—that is, Lotta Svärd represents also the leader’s relationship to himself.^187^ Nursing and Medical Lottas likely mirrored the ‘moral’ and utopian ideal of Nazi leadership and stood proxy for those divine virtues, without which the image of the leader is weak or cannot even take form.^188^ As men could not be seen in isolation, women were placed as present in men’s self-images. The mythic Lottas not only reinforced the modern masculine image of German men but were ultimately precious for Germany because they informed and taught about the Third Reich’s beliefs and values. Medical Lottas were suitable role models, story participants and appurtenances or accessories, as Geertz says, ‘inherited or invented’^189^ by the Third Reich’s political thought, aesthetics and bureaucracy.

## Footnotes

1 Equivalent to head nurse, sister or matron. All names of the Lottas in this article are pseudonyms; this has been done to protect their anonymity and yet to preserve a sense of individuality. Translations from German are mine, unless stated otherwise.

2 Joseph Goebbels, *Die Tagebücher von Joseph Goebbels* (München: K.G. Saur Verlag, 2006), 255.

3 Hilda B.’s Estate (henceforth HE), undated notebook, folder 6, notebooks 1929–1944 (henceforth NB), Satakunta Archive, Satakunta Museum, Finland (henceforth SASM).

4 Katrin Himmler and Michael Wildt, eds, *The Private Heinrich Himmler: Letters of a Mass Murderer*, Thomas S. Hansen and Abby J. Hansen (trans) (New York: St. Martin’s Press, 2016), 152, 294.

5 Jutta Mühlenberg, *Das SS-Helferinnenkorps: Ausbildung, Einsatz Und Entnazifizierung Der Weiblichen Angehörigen Der Waffen-SS 1942–1949* (Hamburg: Hamburger Edition HIS, 2010), 44–45.

6 Elizabeth Harvey, *Women in the Nazi East: Agents and Witnesses of Germanization* (New Haven: Yale University Press, 2003); Elizabeth Harvey, ‘International Networks and Cross-Border Cooperation: National Socialist Women and the Vision of a “New Order” in Europe’, *Politics, Religion & Ideology*, 2012, 13, 141–58.

7 David Stahel, *Operation Barbarossa and Germany’s Defeat in the East* (New York: Cambridge University Press, 2009), 357.

8 David Stahel, *Operation Barbarossa,* 357; Felix Römer, ‘The Wehrmacht in the War of Ideologies: The Army and Hitler’s Criminal Orders on the Eastern Front’, in Alex J. Kay, Jeff Rutherford, and David Stahel, eds, *Nazi Policy on the Eastern Front, 1941: Total War, Genocide, and Radicalization* (Woodbridge, UK: Boydell and Brewer, 2012), 73; Michael Jonas, ‘The Politics of an Alliance: Finland in Nazi Foreign Policy and War Strategy’, in Kinnunen and Kivimäki, eds, *Finland in World War II* (Leiden: Brill, 2012), 93–138.

9 Ville Kivimäki, ‘Introduction: Three Wars and their Epitaphs: The Finnish History and Scholarship of World War II’, Tiina Kinnunen and Ville Kivimäki, eds, *Finland in World War II* (Leiden: Brill, 2012), 1–46.

10 Annika Latva-Äijö, *Lotta Svärdin synty. Järjestö, armeija, naiseus 1918–1928* (Helsinki: Otava, 2004); Maritta Pohls and Annika Latva-Äijö, *Käytännön isänmaallisuutta* (Helsinki: Otava, 2009); Tiina Kinnunen, *Kiitetyt ja parjatut: Lotat sotien jälkeen* (Helsinki: Otava, 2006); Vilho Lukkarinen and Vilho Tervasmäki, *Suomen Lotat: Lotta Svärd -järjestön Historia* (Porvoo, Helsinki, Juva: WSOY, 1981).

11 One master’s thesis—though not focused on medical history—has dealt with Lotta Svärds’ journeys to the Third Reich. Jenna Saarenketo, ‘Suomen lotat Kolmannen valtakunnan vieraina. Lottien kokemuksia palkinto- ja virkistysmatkoilta Saksaan vuosina 1943–1944’ (MA Thesis, University of Turku, 2021).

12 Kiran Klaus Patel, ‘Welfare in the Warfare State: Nazi Social Policy on the International Stage’, *Bulletin of the German Historical Institute London*, 2015, 3–38; Sandrine Kott and Kiran Klaus Patel, eds, *Nazism Across Borders: The Social Policies of the Third Reich and Their Global Appeal* (Oxford: OUP, 2018).

13 Patel, ‘Nazi Social Policy’, 13.

14
*Ibid.*

15 Patel, ‘Nazi Social Policy’, 29; Daniel Hedinger, ‘The Axis at Work? Towards a Transnational History of Japan’s Social and Labour Policy in the 1930s and Early 1940s’, in Sandrine Kott and Kiran Klaus Patel, eds, *Nazism Across Borders: The Social Policies of the Third Reich and Their Global Appeal* (Oxford: OUP, 2018), 194–98; Mats Ingulstad, ‘Under the Hard Law of War: Norwegian Social Reforms under German Influence’, in Sandrine Kott and Kiran Klaus Patel, eds, *Nazism across Borders: The Social Policies of the Third Reich and Their Global Appeal* (Oxford: OUP, 2018), 238–39.

16 Through NSDAP Office of Foreign Affairs, *Nordische Gesellschaft* was led by Alfred Rosenberg.

17
*Lotta-Svärd: Lotta-Svärd yhdistyksen äänenkannattaja* (henceforth *Lotta Svärd*).

18 Kaarle Sulamaa, *Lotat, uskonto ja isänmaa* (Helsinki: SKS, 2009), 154; Suoma Loimaranta-Airila, ‘Lääkintäjaoston lottien koulutus’, *Lotta-Svärd Julkaisu*, 1938, 39, 4.

19 Sybille Steinbacher, ed, *Volksgenossinnen. Frauen in der NS-Volksgemeinschaft* (Göttingen: Wallstein Verlag, 2007), 15–16, 21–24; Thomas Kühne, *The Rise and Fall of Comradeship: Hitler’s Soldiers, Male Bonding and Mass Violence in the Twentieth Century* (Cambridge: Cambridge University Press, 2017), 78, 80, 292–95.

20 Hilde Steppe, ‘Nursing in Nazi Germany’, *Western Journal of Nursing Research*, 1992, 14, 744–53; Kara Dixon Vuic, ‘Wartime Nursing and Power’, in Patricia D’Antonio, Julie A. Fairman and Jean C. Whelan, eds, *Routledge Handbook on the Global History of Nursing NIP* (Abingdon, Oxon: Routledge, 2013), 28–29; Jill Stephenson, *The Nazi Organisation of Women* (London: Taylor & Francis Group, 2012), 113, 192; Claudia Koonz, *Mothers in the Fatherland: Women, the Family, and Nazi Politics* (London: Taylor & Francis Group, 2012), 389.

21 Saarenketo, ‘Suomen lotat’, 20.

22 4 June 1944, HE/NB/SASM.

23 Ingulstad, ‘Norwegian Social Reforms’, 238; Christine Hallett, ‘Guest Editorial: Nursing History and the Articulation of Power’, *Journal of Clinical Nursing*, 2009, 18, 2681–83.

24 Philippe Burrin, *France under the Germans: Collaboration and Compromise*, Janet Lloyd (trans) (New York: New Press, 1996), 460.

25 HE, folder 2, documents concerning decorations 1940–1980, SASM.

26 Ritva Virtanen, ‘Nurses in Finland’s Wartime Medical Service Tasks in the 20th Century’ (PhD thesis, University of Kuopio, 2005), 70–74, 242, 251.

27 Harvey, ‘National Socialist Women’, 141–58.

28 Latva-Äijö, *Lotta Svärdin synty*, 71, 88, 272–81, 293.

29 Mannerheim introduced the concept of ‘white women of Satakunta’ for the white army against Bolshevism to public knowledge. Hilda received numerous decorations from Mannerheim. *Satakunnan Lotta*, 1 January 1930, 10; HE, folder 2, documents concerning decorations 1933–1957, SASM.

30 HE, Undated Welcome Speech to Mannerheim, folder 3, speeches 1940–1942 (henceforth SP), SASM.

31 Undated Speech, HE/SP/SASM. Christiane Berger, ‘Die “Reichsfrauenführerin” Gertrud Scholtz-Klink Zur Wirkung einer nationalsozialistischen Karriere in Verlauf, Retrospektive und Gegenwart’ (PhD thesis, University of Hamburg, 2006), 59, 271.

32
*Hakkapeliitta* (main organ of the Finnish Civil Guard), 1 January 1928, 25; 25 January 1930, 110; 9 November 1937, 1396; Sandrine Kott and Kiran Klaus Patel, ‘Fascist Internationalism: Nazi Social Policy as an Imperial Project—An Introduction’, in Sandrine Kott and Kiran Klaus Patel, eds, *Nazism across Borders: The Social Policies of the Third Reich and Their Global Appeal* (Oxford: OUP, 2018), 23; Daniela Liebscher, ‘Transferring Radicalization? Social Policy Exchanges between Fascism and National Socialism’, in Sandrine Kott and Kiran Klaus Patel, eds, *Nazism across Borders: The Social Policies of the Third Reich and Their Global* Appeal (Oxford: OUP, 2018), 142, 145; Hedinger, ‘The Axis at Work?’, 178.

33
*Hakkapeliitta*, 10 July 1926, 10–11.

34
*Sairaanhoitajatarlehti* (the main organ of Finnish Nurses Association), 1 September 1925, 68; 1 September 1928, 149; 1 February 1935, 40; 1 August 1940, 187; *Lotta Svärd*, 30 November 1936, 302; 30 September 1937, 231; 31 May 1939, 165; 1 November 1942, 402–03.

35 German maternity care was considered as an ideal model. Family Federation of Finland started training home-care workers in 1945. *Sairaanhoitajatarlehti*, 1 November 1941, 293.

36 Arthur Kleinman, *Patients and Healers in the Context of Culture: An Exploration of the Borderland Between Anthropology, Medicine, and Psychiatry* (Berkeley: University of California Press, 1981), 24–26; Clifford Geertz, *Local Knowledge: Further Essays in Interpretive Anthropology* (New York: Basic Books, 2000), 57–58, 69–70; Clifford Geertz, *Negara: The Theatre State in Nineteenth-Century Bali* (Princeton, NJ: Princeton University Press, 1980), 98–120; Clifford Geertz, *The Interpretation of Cultures* (New York, NY: Basic Books, 1973), 314, 334–35, 419, 450.

37
*Lotta Svärd*, 1 June 1943, 187–88.

38 Annette Michel, ‘“Führerinnen” im Dritten Reich. Die Gaufrauenschaftsleiterinnen der NSDAP’, in Sybille Steinbacher, ed, *Volksgenossinnen. Frauen in der NS-Volksgemeinschaft* (Göttingen: Wallstein Verlag, 2007), 117–22.

39 Fanni Luukkonen to Hilda, 8 August 1942, HE, folder 1, Correspondence 1924–1983 (henceforth CORR), SASM.

40
*Ibid.*

41
*Lotta Svärd*, 1 November 1928, 3–5.

42 Tom Mason and Elizabeth Mason-Whitehead, *Thinking Nursing* (Berkshire: McGraw-Hill Education, 2003), 145.

43 Susanne Kreutzer, ‘Conflicting Christian and Scientific Nursing Concepts in West Germany, 1945–1970’, in Patricia D’Antonio, Julie A. Fairman and Jean C. Whelan, eds, *Routledge Handbook on the Global History of Nursing NIP* (Abingdon, Oxon: Routledge, 2013), 155–59.

44 Fanni Luukkonen to Hilda, 8 August 1942, HE/CORR/SASM.

45 George L. Mosse, *The Crisis of German Ideology: Intellectual Origins of the Third Reich* (Madison: University of Wisconsin Press, 2021), 15.

46
*Nordische Gesellschaft*’s journal *Norden* promoted this image. *Norden*, 1 November 1941, 5.

47 Peter Longerich, *Heinrich Himmler*, Jeremy Noakes and Lesley Sharpe (trans) (Oxford: OUP, 2012), 274, 279, 286; Michael H. Kater, *Das ‘Ahnenerbe’ der SS 1935–1945. Ein Beitrag zur Kulturpolitik des Dritten Reiches* (München: Oldenbourg Wissenschaftsverlag, 2006), 18–21, 50, 176–77, 210; Michael H. Kater, *Hitler Youth* (Cambridge, MA: Harvard University Press, 2006), 97.

48 Birgitta Almgren, Jan Hecker-Stampehl and Ernst Piper, ‘Alfred Rosenberg und die Nordische Gesellschaft: Der “nordische Gedanke” in Theorie und Praxis’, *Nordeuropaforum—Zeitschrift für Kulturstudien*, 2008, 2, 51.

49
*Norden*, 1 August 1942, 251.

50
*Lotta-Svärd*, 20 July 1934, 173–76; 14 September 1935, 228–29; 15 October 1936, 249; 30 November 1936, 302; 26 July 1941, 244; 15 November 1941, 350; 1 February 1942, 71.

51
*Lotta Svärd,* 1 June 1943, 188–89.

52 Kater, *Ahnenerbe*, 42, 46; *Lotta Svärd*, 16 May 1935, 16; *Uusi Suomi*, 2 August 1936, 7; *Suomi-Saksa*, 1 June 1942, 5, 16–17; *Hufvudstadsbladet*, 16 November 1946, 4.

53 Undated Speech, HE/SP/SASM.

54
*Ibid.*

55 David S. Reynolds, *Beneath the American Renaissance: The Subversive Imagination in the Age of Emerson and Melville* (Oxford: OUP, 2011), 57–58, 339.

56 Undated Speech, HE/SP/SASM.

57 A telling motto: ‘Do not be important’, appears in the training materials (‘mental/psychological tasks’) of the Lotta Svärd Youth organisation (Little Lottas). See also Elizabeth Harvey, Johannes Hürter, Maiken Umbach and Andreas Wirsching, eds, *Private Life and Privacy in Nazi Germany* (Cambridge: Cambridge University Press, 2019), 30–54.

58 Stephenson, *Nazi Organisation*, 23; Berger, ‘Die “Reichsfrauenführerin”, 69.

59 Steppe, ‘Nursing’, 746; Amy Liane Zroka, ‘Serving the *Volksgemeinschaft*: German Red Cross Nurses in the Second World War’ (PhD thesis, University of California, 2015), 114–15.

60 Zroka, ‘German Red Cross’, 41, 114; Steppe, ‘Nursing’, 748, 752; Dieter Riesenberger, *Das Deutsche Rote Kreuz: eine Geschichte, 1864–1990* (Paderborn: Schöningh, 2002).

61 Berger, ‘Die “Reichsfrauenführerin”’, 8, 267, 274; Zroka, ‘German Red Cross’, 493–94; Massimiliano Livi, *Gertrud Scholtz-Klink: Die Reichsfrauenführerin. Politische Handlungsräume und Identitätsprobleme der Frauen im Nationalsozialismus am Beispiel ‘der Führerin aller deutschen Frauen*’ (Münster: LIT Verlag, 2005), 221, 224.

62
*Allgemeine Zeitung*, 18 July 1924, 2.

63
*Lotta Svärd*, 20 July 1934, 175.

64 Almgren, Hecker-Stampehl and Piper, ‘Alfred Rosenberg’, 13.

65 Kater, *Ahnenerbe*, 18, 176–79, 210; Kater, *Hitler Youth*, 95–97; George L. Mosse, *The Image of Man: The Creation of Modern Masculinity* (New York: OUP, 1998), 169, 176; Ingulstad, ‘Norwegian Social Reforms’, 238–39.

66
*Jeversches Wochenblatt: Friesisches Tageblatt*, 5 May 1934, 8.

67
*Ibid.*

68
*Frauenzeitung der Badischen Presse*, 12 April 1934, 1.

69 Steinbacher, *Volksgenossinnen*, 24–25; Sybille Steinbacher, ‘Differenz der Geschlechter? Chancen und Schranken fur die “Volksgenossinnen”’, in Frank Bajohr and Michael Wildt, eds, *Volksgemeinschaft: Neue Forschungen zur Gesellschaft des Nationalsozialismus* (Frankfurt/M: Fischer Taschenbuch Verlag, 2009), 94–104.

70
*Lotta-Svärd*, 16 September 1929, 201–02; 30 October 1930, 236; 15 November 1937, 273–75; 20 January 1941, 2; 1 October 1942, 354; 1 September 1943, 276–77, 291.

71
*Lotta Svärd*, 25 March 1941, 49.

72
*Lotta Svärd*, 20 October 1941, 337.

73 Mosse, *The Image of Man*, 53–54, 74.

74
*Lotta Svärd*, 20 October 1941, 337.

75
*Lottatyttö*, 1 September 1943, 13.

76
*Nordlicht*, 1 January 1941, 24–27.

77 Mosse, *The Image of Man*, 54; Kühne, *Comradeship*, 86; Steinbacher, *Volksgenossinnen*, 24.

78 Steinbacher, *Volksgenossinnen*, 15–16, 21–24; Steinbacher, ‘Differenz der Geschlechter?’, 94–104.

79 Geertz, *Local Knowledge*, 129; Geertz, *The Theatre State*, 108, 120.

80 Mosse, *The Image of Man*, 154; George L. Mosse, ‘Fascist Aesthetics and Society: Some Considerations’, *Journal of Contemporary History*, 1996, 31, 245–52.

81 HE, Undated and Unnamed guestbook, folder 6, memory and guestbooks, 1932–1958 (henceforth GB), SASM.

82 Michel, ‘Führerinnen’, 121.

83 31 May 1944, HE/NB/SASM.

84 Riesenberger, *Das Deutsche Rote Kreuz*, 267; Zroka, ‘German Red Cross’, 89–90.

85 31 May 1944, HE/NB/SASM.

86
*Ibid.*

87 Longerich, *Himmler*, 286.

88 5 July 1944, HE/NB/SASM.

89 1 June 1944, HE/NB/SASM.

90 Undated, HE/NB/SASM.

91 30 June 1944, HE/NB/SASM.

92 3 June 1944, HE/NB/SASM.

93 16 June 1944, HE/NB/SASM.

94 Michael G. Kenny, ‘A Darker Shade of Green. Medical Botany, Homeopathy, and Cultural Politics in Interwar Germany’, *Social History of Medicine*, 2002, 15, 500–01.

95 Maiken Umbach, ‘(Re-)Inventing the Private under National Socialism’, in Elizabeth Harvey *et al.*, eds, *Private Life and Privacy in Nazi Germany* (Cambridge: Cambridge University Press, 2019), 116–18.

96 Undated, June 1944, HE/GB/SASM.

97 19 July 1944, HE/CORR/SASM.

98 30 July 1944, HE/CORR/SASM.

99
*Ibid.*

100 Undated, HE/GB/SASM.

101 9 June 1944, HE/NB/SASM.

102 23 June 1944, HE/NB/SASM.

103
*Ibid*.

104 7 June 1944, HE/NB/SASM.

105 Enno Georg, *Die Wirtschaftlichen Unternehmungen der SS* (Stuttgart: Deutsche Verlags-Anstalt, 1963), 102.

106 Josef Henke, *Persönlicher Stab Reichsführer-SS: Bestand NS 19*, part 2 (Koblenz: Bundesarchiv, 1997), 493–95.

107 Undated, June 1944; 2 July 1944; 23 July 1944, HE/GB/SASM.

108 Undated, July 1944, HE/GB/SASM.

109 Mühlenberg, *Das SS-Helferinnenkorps*, 40–45.

110 2 July 1944, HE/GB/SASM.

111
*Ibid.*

112 30 June 1944, HE/GB/SASM.

113 7 July 1944, HE/GB/SASM.

114 Longerich, *Himmler*, 19, 23, 53, 77, 105, 467–68.

115 2 July 1944, HE/GB/SASM.

116 15, 16, 18, 22 and 28 June 1944, HE/NB/SASM.

117 Kara Dixon Vuic, *Officer, Nurse, Woman: The Army Nurse Corps in the Vietnam War* (Baltimore: Johns Hopkins University Press, 2009), 149–51.

118 Berger, ‘Die “Reichsfrauenführerin”’, 73.

119 Kreutzer, ‘Christian and Scientific Nursing’, 151.

120 Undated, HE/GB/SASM; Berger, ‘Die “Reichsfrauenführerin”’, 73–75.

121 The Christmas carol ‘Leise rieselt der Schnee’ was composed by Eduard Ebel. Undated, HE/GB/SASM.

122 Berger, ‘Die “Reichsfrauenführerin”’, 74–75.

123 Kreutzer, ‘Christian and Scientific Nursing’, 158.

124 Mühlenberg, *Das SS-Helferinnenkorps,* 88.

125 Virtanen, ‘Finland’s Wartime Medical Service’, 229–30, 255.

126 16 July 1944, HE/GB/SASM.

127 23 July 1944, HE/GB/SASM.

128 Zroka, ‘German Red Cross’, 267–69.

129 Undated, July 1944, HE/GB/SASM.

130 Virtanen, ‘Finland’s Wartime Medical Service’, 138, 159–60, 187–88.

131 Marianne Junila, *Coexistence Between the Finnish Civilian Population and the German Troops in Northern Finland in 1941–1944* (Helsinki: SKS, 2000), 257; Minna Elomaa-Krapu, ‘Willingness to Nursing and Sense of Duty: The Finnish Volunteer Nurses’ Experiences of Education and Military Nursing During the World War II in Finland’ (PhD thesis, Tampere University, 2015), 19, 41, 49.

132 HE, Undated Presentation, folder 3, Speeches 1940–1942 (henceforth Presentation), SASM.

133 Nicole Kramer, *Volksgenossinnen an der Heimatfront: Mobilisierung, Verhalten, Erinnerung* (Göttingen: Vandenhoeck & Ruprecht, 2011).

134 Christine E. Hallett, ‘The Personal Writings of First World War Nurses: A Study of the Interplay of Authorial Intention and Scholarly Interpretation’, *Nursing Inquiry,* 2007, 14, 320–21.

135
*Ibid.*

136 Presentation, 18, HE/SP/SASM.

137 HE, Undated Guestbook of Elisa, folder 6, memorybooks and guestbooks 1932–1958, SASM.

138 Presentation, 6–7, HE/SP/SASM.

139
*Ibid.*

140 Virtanen, ‘Finland’s Wartime Medical Service’, 138.

141 Presentation, 14, HE/SP/SASM.

142 Presentation, 19, HE/SP/SASM.

143 Presentation, 18–19, HE/SP/SASM.

144 Presentation, 14, HE/SP/SASM; Elomaa-Krapu, ‘Willingness to Nursing’, 143.

145 Presentation, 7–8, 13–14, HE/SP/SASM.

146 Presentation, 15–16, HE/SP/SASM.

147 Presentation, 15, HE/SP/SASM.

148 Gerhard B. Kuntscher, ‘The Kuntscher Method of Intramedullary Fixation’, *The Journal of Bone and Joint Surgery*, 1958, 40, 17–26.

149 Küntscher returned to give a lecture at the invitation of the University of Helsinki in 1964 and participated in a congress in Helsinki organised by the International College of Surgeons in 1965. *Hufvudstadsbladet*, 28 November 1943, 7; 17 March 1964, 2; 19 June 1965, 14.

150
*Kansa taisteli*, 15 November 1982, 364–66.

151 Presentation, 13, HE/SP/SASM.

152 Presentation, 8–9, HE/SP/SASM.

153 Presentation, 1, 8, HE/SP/SASM; Virtanen, ‘Finland’s Wartime Medical Service’, 118.

154 Christine E. Hallett and Alison S. Fell, ‘Introduction: New Perspectives on First World War Nursing’, in Alison S. Fell, and Christine E. Hallett, eds, *First World War Nursing: New Perspectives* (London: Taylor & Francis Group, 2013), 18–19.

155 Christine E. Hallett, ‘“Emotional Nursing”: Involvement, Engagement, and Detachment in the Writings of First World War Nurses and VADs’, in Alison S. Fell and Christine E. Hallett, eds, *First World War Nursing*, 182, 191–93.

156 Kühne, *Comradeship*, 86.

157 Presentation, 4–6, HE/SP/SASM.

158 Presentation, 12–13, HE/SP/SASM.

159 Zroka, ‘German Red Cross’, 220–21.

160 Presentation, 10, HE/SP/SASM. See also Kaisa-Maria Peltokorpi, ‘On elettävä kun koska tahansa voi kuolla. Lottien selviytyminen sodassa 1939–1945’ (PhD thesis, University of Lapland, 2012), 241.

161 Presentation, 10, HE/SP/SASM.

162
*Ibid.*

163 Charles Edward Ryan, *With an Ambulance during the Franco-German War: Personal Experiences and Adventures With Both Armies, 1870–1871* (London: Aberdeen University Press, 1896), 169.

164 Presentation, 11, HE/SP/SASM.

165 Presentation, 10–11, HE/SP/SASM.

166
*Ibid.*

167
*Ibid.*

168 Vuic, *Officer, Nurse, Woman*, 147–49.

169 Presentation, 11–12, HE/SP/SASM.

170
*Ibid.*

171 Steinbacher, ‘Differenz der Geschlechter?’, 94–104.

172 Kühne, *Comradeship*, 146, 292–95.

173 HE, folder 2, documents concerning decorations 1940–1980, SASM.

174 Fanni Luukkonen to Hilda, 28 July 1945, HE/CORR/SASM.

175
*Ibid.*

176 Kühne, *Comradeship*, 86.

177 2 July 1944, HE/NB/SASM.

178 Patel, ‘Nazi Social Policy’, 13–20; Kott and Patel, ‘Fascist Internationalism’, 12.

179 Kott and Patel, ‘Fascist Internationalism’, 13–14.

180 Konrad H. Jarausch, ‘Organic Modernity: National Socialism as Alternative Modernism’, in Shelley Baranowski, Armin Nolzen and Claus-Christian W. Szejnmann, eds, *A Companion to Nazi Germany* (Hoboken, NJ/Chichester: Wiley-Blackwell, 2018), 33–46.

181
*Lotta Svärd*, 20 July 1934, 173.

182 Patel, ‘Nazi Social Policy’, 13.

183 Patel, ‘Nazi Social Policy’, 23–24.

184 Kater, *Ahnenerbe*, 21–23.

185
*Norden*, 1 August 1942, 251.

186 Geertz, *Local Knowledge*, 129, 134.

187 Geertz, *The Theatre State*, 108, 129; Geertz, *The Interpretation*, 417, 419; Geertz, *Local Knowledge*, 129, 143.

188 Geertz, *The Theatre State*, 131.

189 Geertz, *Local Knowledge*, 124; Geertz, *The Interpretation*, 127, 331.

